# Multiple Kernel Based Region Importance Learning for Neural Classification of Gait States from EEG Signals

**DOI:** 10.3389/fnins.2017.00170

**Published:** 2017-04-03

**Authors:** Yuhang Zhang, Saurabh Prasad, Atilla Kilicarslan, Jose L. Contreras-Vidal

**Affiliations:** ^1^Noninvasive Brain-Machine Interface Systems Lab, Department of Electrical and Computer Engineering, University of HoustonHouston, TX, USA; ^2^Hyperspectral Image Analysis Lab, Department of Electrical and Computer Engineering, University of HoustonHouston, TX, USA

**Keywords:** brain machine interface (BMI), neural classification, electroencephalography (EEG), machine learning, multiple kernel learning

## Abstract

With the development of Brain Machine Interface (BMI) systems, people with motor disabilities are able to control external devices to help them restore movement abilities. Longitudinal validation of these systems is critical not only to assess long-term performance reliability but also to investigate adaptations in electrocortical patterns due to learning to use the BMI system. In this paper, we decode the patterns of user's intended gait states (e.g., stop, walk, turn left, and turn right) from scalp electroencephalography (EEG) signals and simultaneously learn the relative importance of different brain areas by using the multiple kernel learning (MKL) algorithm. The region of importance (ROI) is identified during training the MKL for classification. The efficacy of the proposed method is validated by classifying different movement intentions from two subjects—an able-bodied and a spinal cord injury (SCI) subject. The preliminary results demonstrate that frontal and fronto-central regions are the most important regions for the tested subjects performing gait movements, which is consistent with the brain regions hypothesized to be involved in the control of lower-limb movements. However, we observed some regional changes comparing the able-bodied and the SCI subject. Moreover, in the longitudinal experiments, our findings exhibit the cortical plasticity triggered by the BMI use, as the classification accuracy and the weights for important regions—in sensor space—generally increased, as the user learned to control the exoskeleton for movement over multiple sessions.

## 1. Introduction

Brain Machine Interface (BMI) systems have attracted extensive attention in the past decade, because of their potential in improving human life, especially for those who are affected by motor disabilities. Since gait deficits are commonly associated with spinal cord injuries (SCI), limb loss, and neurodegenerative diseases, there is a need to investigate innovative therapies to restore gait in such patients. Exoskeletons have become prominent tools for the rehabilitation of SCI and stroke patients (Sale et al., 2012; Venkatakrishnan et al., 2014). BMIs have been deployed to infer the user's intent from his/her brain activity to generate output signals to control powered exoskeletons for upper and lower limb rehabilitation (Noda et al., 2012; Contreras-Vidal and Grossman, 2013; Kilicarslan et al., 2013; French, 2014; Venkatakrishnan et al., 2014). In Presacco et al. (2011), Presacco et al. showed decoding of gait kinematics during treadmill walking from EEG of able-bodied subjects with accuracies comparable to that from a similar study in non-human primates with electrodes implanted in their brains (Fitzsimmons et al., 2009). Further, in Kilicarslan et al. (2013), a paraplegic subject's motion intentions were accurately decoded using Locality Fisher Discriminant Analysis and a Gaussian Mixture Model (LFDA-GMM) for two different gait tasks (i.e., repeated walking-turning right-turning left motions and sit-rest-stand motions). The model enabled the closed-loop EEG-based BMI system to control a robotic exoskeleton (NeuroREX) in real-time, resulting in independent walking for the paraplegic user.

To control a device via BMI, different brain activity patterns produced by a user need to be accurately identified by a neural interface system and translated into appropriate commands (Contreras-Vidal et al., 2015). Discrete decoding (neural classification) of intent from EEG signals can be considered as a pattern recognition problem, and advanced machine learning techniques are needed to accurately translate the brain electrical activities to meaningful control commands. Many machine learning methods [e.g., linear discriminant analysis (LDA), support vector machine (SVM), Bayesian classifiers] have been applied for classifying EEG signals in different BMI applications (Kilicarslan et al., 2013; Niazi et al., 2013; Leamy et al., 2014; Lew et al., 2014; Hortal et al., 2015; Jiang et al., 2015). However, most of them serve as a “black box” in that we do not know how the brain activity changes during long-term BMI use nor how the brain regions contribute to the classification process while people perform different tasks. The human brain consists of over 100 billion cells, typically divided into regions by neuroanatomists. Different regions have their specific functionalities while coordinating together to accomplish everyday tasks. Moreover, the specific contributions of brain regions to classification may change due to learning a BMI. Therefore, it is important to identify and track these changes to increase our understanding of brain function, BMI learning and performance. In that context, the hypothesis of this research is that different brain regions contribute differentially to BMI learning and control of robot assisted lower-limb movements—we are interested in learning the importance of these regions for neural classification of gait states.

Kernel learning methods have been effectively applied for many machine learning problems, including feature selection, data regression and classification for EEG signals (Garrett et al., 2003; Lal et al., 2004; Lotte et al., 2007). SVM is one of the most popular kernel methods for pattern recognition. However, a problem with using the standard SVM in BMI applications is that it provides no insight about the importance of distinct features, and thus has little knowledge about the biophysical properties of relevant features used in decoding/classification. Multiple kernel learning (MKL), which makes use of a combination of basis kernels to represent different types of features or data, have been shown to outperform traditional single-kernel machines in different aspects (Sonnenburg et al., 2006; Tian et al., 2012; Samek et al., 2013; Li et al., 2014). The main advantage of using MKL over SVM is that MKL can simultaneously learn the classifier and the optimal weights for basis kernels. In this paper, we investigate and make use of this property to simultaneously decode gait states from multi-channel EEG signals and learn the relative importance of different scalp brain areas. Particularly, we build a composite kernel based on a linear combination of basis kernels, in which each basis kernel can be represented by a group of electrodes corresponding to selected regions of interest (ROIs), and consequently contribute unique biophysical information.

The primary goal of this research is to show the feasibility of simultaneously classifying the pattern of user's internal gait states (e.g., stop, walk, turn left, turn right) from the EEG signals and learning the relative importance of different scalp brain areas. Previous studies have shown that low delta band (0.1–2 Hz) EEG contains intended movement-related information for decoding the kinematics of lower limb or gait states (Presacco et al., 2011, 2012; Jorquera et al., 2013; Kilicarslan et al., 2013; Bulea et al., 2014; Luu et al., 2016). For example, in Presacco et al. (2011), Presacco et al. (2012), and Luu et al. (2016), it was shown that delta band EEG contains information about gait movement kinematics that can be decoded using Wiener or Kalman filters. In Kilicarslan et al. (2013), Jorquera et al. (2013), and Bulea et al. (2014), it was shown that movement-type (e.g., “stop,” “go,” etc.) classifiers can be designed based on delta band EEG signals. Another study (Velu and de Sa, 2013) showed that features corresponding to frequencies less than 2 Hz were the most heavily weighted during single trial classification of walking and pointing direction. Inspired by the above findings, in this study, we utilize delta band (0.1–2 Hz) EEG to build our basis kernels (feature matrices) for neural classification of gait states.

The other goal of the research is to compare the brain regions employed for classifying movement intents from able-bodied subjects and individuals with spinal cord injury (SCI) given differences in neural activity across these populations. Studies have shown that SCI can cause widespread and sustained brain inflammation that leads to progressive loss of brain cells in key brain regions with associated cognitive problems (Wu et al., 2014a,b). Cramer et al. have found that in patients with complete SCI, many features of normal motor system function are preserved, however, the volume and patterns of activation and the modulation of function with change in task are abnormal and absent, respectively, in patients with SCI (Cramer et al., 2005). In this preliminary study, we collected EEG data from a SCI volunteer over multiple sessions to compare the classification results with an able-bodied subject on the important brain regions during learning.

The remainder of the paper is organized as follows. Section 2 introduces our methodology for region importance learning, including experimental protocol, data acquisition, processing and analysis. In particular, we introduce the MKL algorithm and how we apply it to learn the importance of different brain regions. In section 3, we validate the efficacy of the proposed work via experiments using four-class single session and two-class longitudinal EEG data. Section 4 presents our discussions from analyzing the experimental results. Finally, concluding remarks are provided in Section 5.

## 2. Methodology

### 2.1. Experimental protocols and tasks

The experimental protocols were approved by the Institutional Review Board of the University of Houston. After giving written informed consent, an able-bodied subject and an SCI subject (both male) were fitted with a wearable powered exoskeleton (REX, REX Bionics Ltd, New Zealand) and an EEG-based BMI (Kilicarslan et al., 2013). For data collection, users were asked to perform motor imagery of locomotive movements while following and completing a path marked on the ground with the robot controlled by an operator remotely. This allowed synchronized motion and EEG data while securing user engagement. There were two tasks in this research. Task 1 was a four-class, single session task in which the subjects performed different movements, i.e., walking forward, turning right, turning left and stop, following the marked path on the ground. In Task 2, subjects only executed walking and stop motions according to audible beep instructions. Each trial contained at least 10 stop-to-walk or walk-to-stop transitions. The subjects were trained over multiple sessions in a 30 days period to control the exoskeleton to perform these motions.

### 2.2. Data acquisition and processing

**Figure 1:**
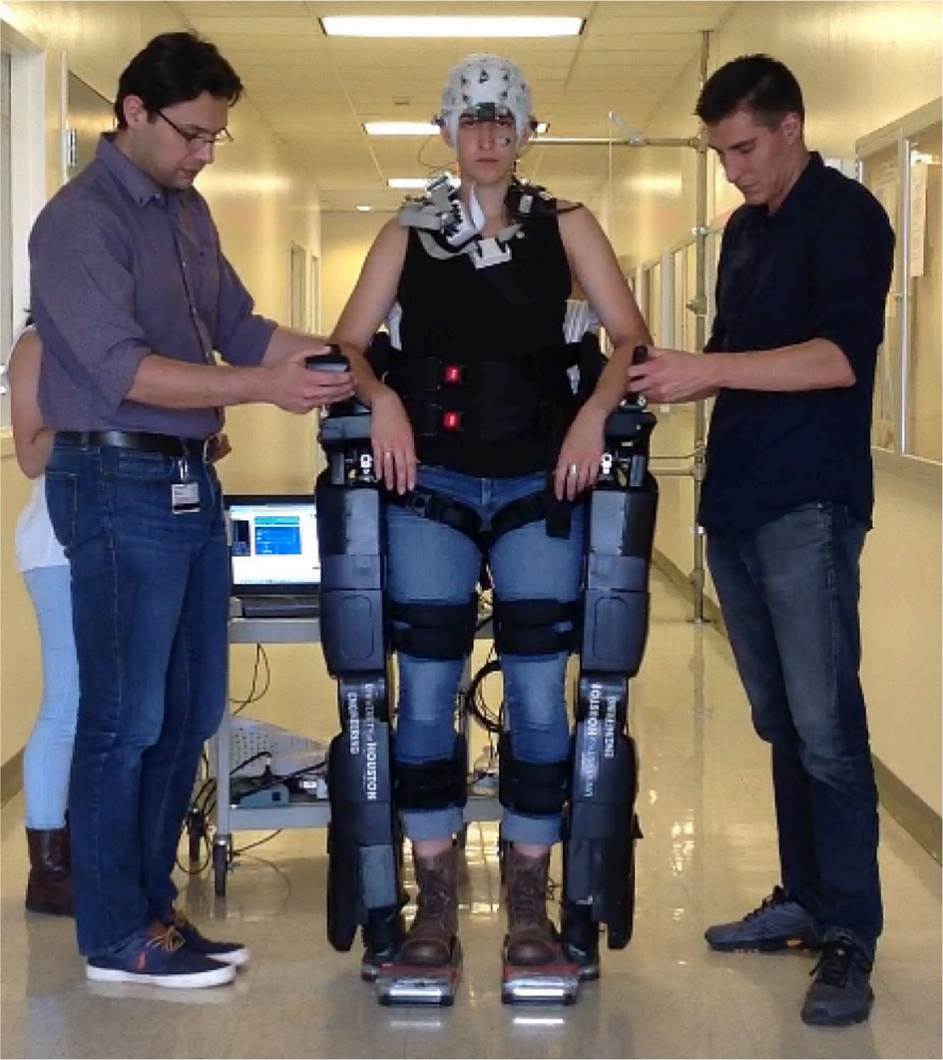
**A volunteer controlling NeuroRex via the EEG BMI system**.

Multichannel active-electrode EEG (64 channels) was recorded by combining two 32-channel amplifiers (actiCap system, Brain Products GmbH, Germany). The electrodes were placed and labeled in accordance with the extended 10–20 international system. A wireless interface (MOVE system, Brain Products GmbH, Germany) was used to transmit data (sampled at 100 Hz) to the host PC. Figure 1 shows a volunteer controlling NeuroRex via the EEG BMI system.

We took a careful approach in regard to potential motion artifacts aiding decoding. First, we used good engineering measurement practices (Nathan and Contreras-Vidal, 2015), including EEG cap set-up and medical-grade mesh to fixate individual electrode wirings that can induce motion artifacts; second, we deployed a wireless active-electrode EEG system to increase the signal to noise ratio (signals are amplified directly at the electrode location) and help mitigate motion artifacts; third, we have shown that the delta band EEG contains negligible motion artifacts at the gait speeds tested in the study (Nathan and Contreras-Vidal, 2015); fourth, we applied the Artifact Subspace Reduction (ASR, an automated artifact rejection method Mullen et al., 2013; Bulea et al., 2014) and compared classification accuracies with and without ASR, to assess the potential effects of motion artifacts but did not find significant changes on classification accuracies suggesting that motion artifacts, if any, did not affect decoding. The acquired data were then filtered in the 0.1–2 Hz range using a second order Butterworth filter and standardized (z-score) in a data preprocessing step.

### 2.3. Region importance learning framework

**Figure 2:**
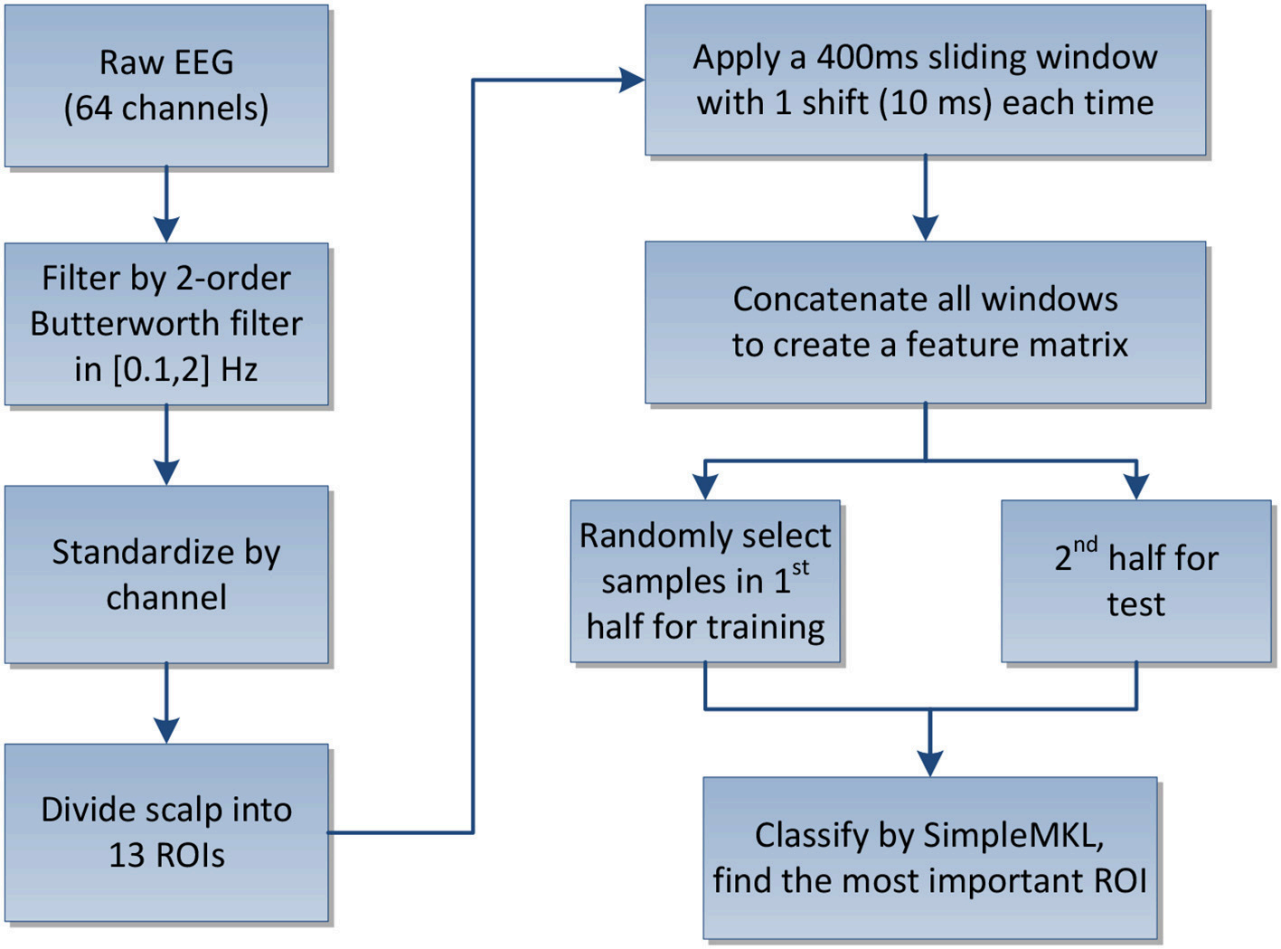
**Flowchart of the region importance learning framework**.

We conducted separate experiments of the above two tasks to interpret the use of kernel weights in MKL as an indicator of the region importance in classification of user's movement intention from EEG signals. After the signals were pre-processed, 64 channels were divided into 13 ROIs as described in Section 2.4.1. The features were then extracted by applying a 400 ms sliding window on each channel with 1 shift (10 ms) each time to acquire the amplitude modulations and concatenated as a feature matrix. To better meet the data process in real world, we divided the labeled samples into two halves for supervised learning. We randomly select 500 samples from the first half of the labeled samples for training, and the remaining half were used for testing and evaluation. The testing process was repeated 10 times and the metric for evaluating the classification results is the average overall accuracy (OA). The flowchart of the proposed framework is shown in Figure 2.

### 2.4. Learning and classification methods

#### 2.4.1. Brain scalp regions

The analysis and interpretation of EEG measurements depend upon the correspondence of electrode scalp coordinates to structural and functional regions of the brain (Giacometti et al., 2014; Gentili et al., 2015). For example, Giacometti et al. (2014) showed that EEG electrode proximity maps intersect with EEG sensitivity maps of the human brain, allowing the use of proximity maps to inform the cortical origin of scalp recordings. Furthermore, intersection of structural and functional regions of the brain with cortical proximity parcellations can be used to show the correspondences between scalp electrode coordinates and potential regions of interest in the human cortex (Giacometti et al., 2014).

**Figure 3:**
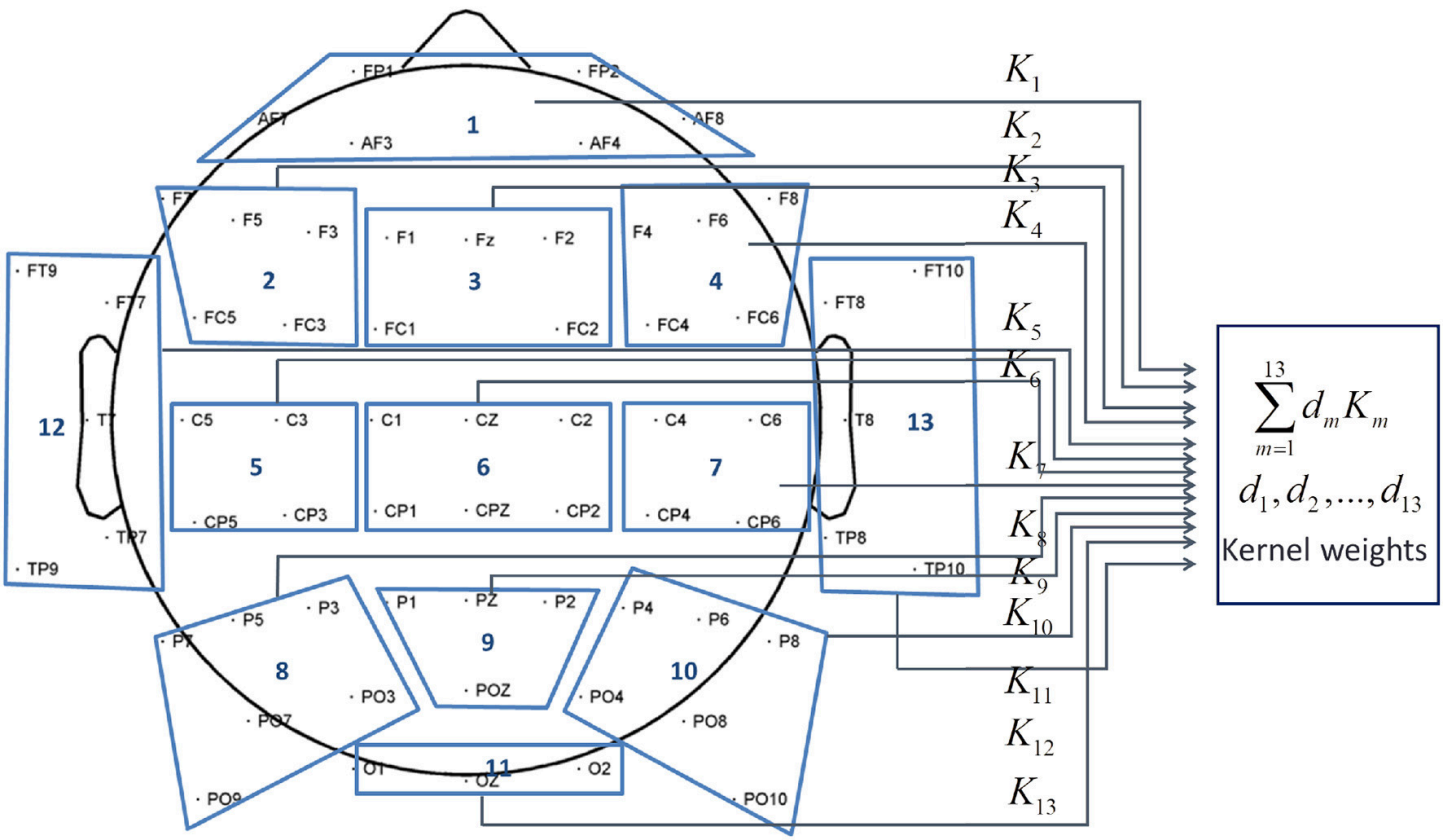
**Scalp regions of interest (ROIs)**.

In this research, we investigate the importance of brain areas for the lower-limb movement neural classification task for both able-bodied and SCI subjects. Specifically, the brain scalp is divided into 13 topographical regions of interest (ROIs) (Kranczioch et al., 2008; Gobel et al., 2011), which are anterior frontal (AF), left fronto-central (LFC), midline fronto-central (MFC), right fronto-central (RFC), left centro-parietal (LCP), midline centro-parietal (MCP), right centro-parietal (RCP), left parieto-occipital (LPO), middle parieto-occipital (MPO), right parieto-occipital (RPO), left temporal (LT), right temporal (RT) and Occipital (O). Figure 3 and Table 1 show the partition of the scalp and the name for each ROI.

**Table 1:** **Scalp ROI names**.

**Index**	**ROI name**	**Index**	**ROI name**
1	Anterior Frontal (AF)	8	Left Parieto-Occipital (LPO)
2	Left Fronto-Central (LFC)	9	Middle Parieto-Occipital (MPO)
3	Midline Fronto-Central (MFC)	10	Right Parieto-Occipital (RPO)
4	Right Fronto-Central (RFC)	11	Left Temporal (LT)
5	Left Centro-Parietal (LCP)	12	Right Temporal (RT)
6	Midline Centro-Parietal (MCP)	13	Occipital (O)
7	Right Centro-Parietal (RCP)		

#### 2.4.2. Kernel-based learning methods foundation

Kernel-based learning methods have been widely applied for various machine learning tasks. The reason for its popularity is that it easily extends the linear classifier to nonlinear decision surfaces using the “kernel trick.” All the kernel methods make use of the “kernel trick” to map the data *X* = {x_1_, x_2_, …, x_*N*_} from the input space to a higher dimensional feature space H (i.e., Reproducing Kernel Hilbert Space (RKHS)) as $\Phi :{\mathbb{R}}^{d}\to H,\;\text{x}\to \Phi \left(\text{x}\right)$, so that the original non-linear data are linear separable in such feature space. The kernel mapping is defined as:

(1) $$K({\text{x}}_{i},{\text{x}}_{j})=\langle \Phi ({\text{x}}_{i}),\Phi ({\text{x}}_{j})\rangle ,$$

where 〈·, ·〉 is the inner product of two vectors.

SVM is one of the most popular kernel-based classifier (Vapnik and Vapnik, 1998). The underlying principle of SVM is to simultaneously minimize the empirical classification error and maximize the geometric margin of the linear separation surface. The optimization problem for SVM classification is formulated as:

(2) $$\begin{array}{l} \underset{w,\xi_{i},b}{\min}J(\text{w},\xi_{i},b)=\frac{1}{2}{\left\|\text{w}\right\|}^{2}+C\sum_{i\;=\;1}^{N}\xi_{i}\\ \text{s}.\text{t}.\{\begin{array}{c} y_{i}\left(\langle \text{w},\Phi ({\text{x}}_{i})\rangle +b\right)\geq 1-\xi_{i}\\ \xi_{i}\geq 0,\forall i=1,2,\cdots ,N \end{array}, \end{array}$$

where *C* is a constant which controls the balance between the margin and empirical loss, ξ_*i*_ are slack variables which measure the degree of misclassification, and ||**w**||^2^ is inversely related to the margin to the hyperplane.

In most kernel-based learning methods, performance is greatly affected by the choice of kernel function and related kernel hyper-parameters. The standard SVM only utilizes a single kernel function with fixed parameters, which necessitates model selection for good classification performance. Besides, using a fixed kernel may be suboptimal, since different sources of data may have different representations of the phenomena of interest, and hence the similarity should not be measured via the same kernel function.

#### 2.4.3. Multiple kernel learning (MKL)

In recent works, MKL has been shown to outperform traditional single-kernel SVMs in many cases, especially for classification and feature fusion problems (Sonnenburg et al., 2006; Tian et al., 2012; Samek et al., 2013; Li et al., 2014; Zhang et al., 2015). In this paper, we employ MKL to infer information about electrode relevance by observing the kernel weights learned from training the machine for classification. Each “group” of features is assigned a basis kernel, and the linear combination of all basis kernels is optimized through gradient descent on the SVM objective function. The optimization of multiple kernels works as a feature selector providing a weighted ranking of the importance of its components.

We consider the above 13 ROIs as generating a 13-source input. For a specific source *p*, the combined kernel function *K* between two samples ${\text{x}}_{i}^{p}$ and ${\text{x}}_{j}^{p}$ can be represented as

(3) $$\begin{array}{l} K({\text{x}}_{i}^{p},{\text{x}}_{j}^{p})=\sum_{m\;=\;1}^{M}d_{m}K_{m}({\text{x}}_{i}^{p},{\text{x}}_{j}^{p})\\ \text{s}.\text{t}.\;d_{m}\geq 0,\text{and}\sum_{m\;=\;1}^{M}d_{m}=1\;, \end{array}$$

where *M* is the number of candidate basis kernels representing different kernel parameters, *K*_*m*_ is the *m*-th basis kernel and *d*_*m*_ is the weight for it. Weights can be estimated through cross-validation, which is computationally demanding when the number of basis kernels (i.e., feature sets or data sources) is large. An alternative strategy, which we adopt in this work, is based on the SimpleMKL algorithm (Rakotomamonjy et al., 2008). It optimizes the weights automatically in a learning problem by utilizing the gradient descent approach. Based on the SVM optimization problem, the SimpleMKL learning problem is expressed as:

(4) $$\begin{array}{l} \underset{d}{\min}J(d),\text{ s}.\text{ t}.\;d_{m}\geq 0,\text{and}\sum_{m\;=\;1}^{M}d_{m}=1\\ J(d)=\{\begin{array}{c} \underset{w,b,\xi }{\min}\frac{1}{2}\sum_{m\;=\;1}^{M}\frac{1}{d_{m}}{\left\|{\text{w}}_{m}\right\|}^{2}+C\sum_{i\;=\;1}^{N}\xi_{i}\\ \begin{array}{l} \text{s}.\text{t}.\;y_{i}\left(\sum_{m\;=\;1}^{M}\langle w_{m},\Phi_{m}({\text{x}}_{i}^{p})\rangle +b\right)\geq 1-\xi_{i}\\ \xi_{i}\geq 0,\forall i=1,2,\cdots ,N\;, \end{array} \end{array} \end{array}$$

where $\Phi_{m}\left({\text{x}}_{i}^{p}\right)$ is the kernel mapping function of $x_{i}^{p}$, *w*_*m*_ is the weight vector of the *m*^*th*^ decision hyperplane, *C* is the regularization parameter controlling the generalization capabilities of the classifier, and ξ_*i*_ is a positive slack variable.

The objective function is a constrained optimization problem, which can be transformed into a dual form *L*(α_*i*_, α_*j*_) using Lagrange multipliers α_*i*_, α_*j*_. Then the kernel weight *d*_*m*_ can be optimized by updating it along the gradient descent direction of *L*(α_*i*_, α_*j*_) as d ← d + γD, where γ is the step length, *D* is the descent direction of *L*(α_*i*_, α_*j*_), and $\text{d}={\left[d_{1},d_{2},\cdots \;,d_{M}\right]}^{\text{T}}$ is the kernel weight vector. Following this optimization procedure and after several iterations, SimpleMKL provides the optimal kernel weight for each basis kernel that indicates the importance of a particular brain region in classification of gait states.

### 2.5. Parameter settings

In the experiments, RBF kernels defined as $K\left(x_{i},x_{j}\right)=\exp\left(-\frac{{\left||x_{i}\;-\;x_{j}|\right|}^{2}}{2\sigma^{2}}\right)$ were used with relative width parameter σ. In the multiple kernel setting, we did not select a specific kernel parameter; instead, we defined a set of different values as candidate input parameters. We can build several basis kernels with different values of σ for each source of input, however, the number of parameters should be kept small to reduce the computational complexity and memory requirements. In particular, four basis kernels with σ = [0.1, 0.5, 1, 1.5] were considered for all sources. This range of values was found to be reasonable after applying kernel alignment (Shawe-Taylor and Kandola, 2002) using cross-validation. The penalty parameter was then selected by cross-validation in the range of [2^−1^, …, 2^15^]. For further information of MKL experimental settings, we refer readers to Zhang et al. (2015). All the experiments were implemented in Matlab R2014a using the SimpleMKL toolbox (Rakotomamonjy, 2008).

## 3. Results

### 3.1. Four-class, single session classification results

**Figure 4:**
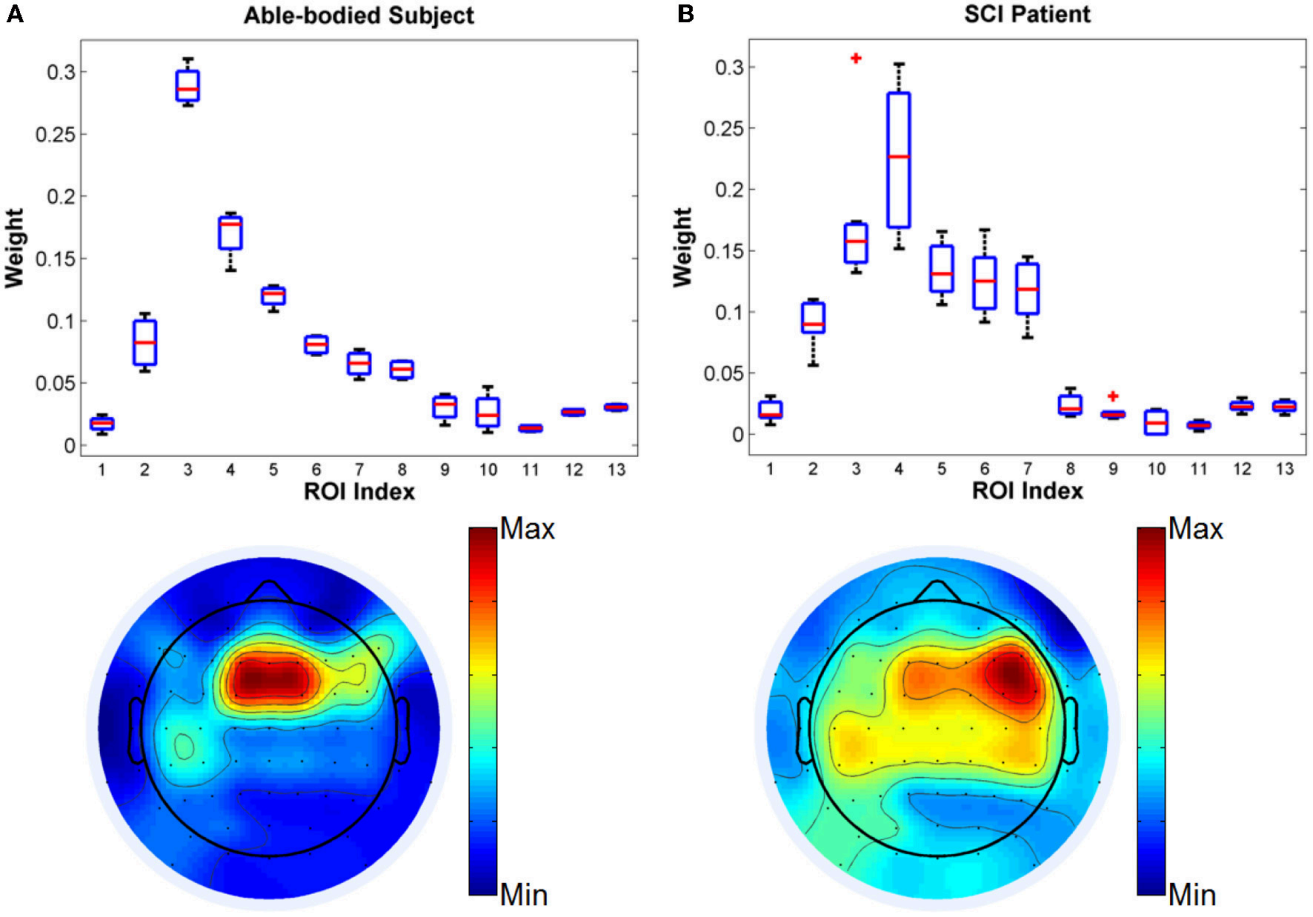
**Comparison of kernel weights for different ROIs from (A)** able-bodied subject and **(B)** SCI subject in Task 1.

First, we compare the kernel weights optimized by SimpleMKL algorithm for defined ROIs from the able-bodied subject and the SCI subject in a four-class task. Four motion classes for classification are walking forward, turning left, turning right and stop. The boxplots and topoplots of optimized kernel weights for different ROIs are shown in Figure 4. The average classification accuracies were 74.5% and 68.4% for the able-bodied and the SCI subject, respectively.

From the results, it is observed that the fronto-central scalp regions (MFC, RFC) have the highest weights among all ROIs, which included scalp areas associated with the motor planning and the lower-limb neural representation (Leeb et al., 2013). Interestingly, for the able-bodied subject, the MFC ROI showed the highest relevance to gait decoding with RFC being the closest area in importance. In contrast, in the case of the SCI subject, the order of importance was reversed, with RFC showing the highest relevance followed by MFC. We also note that for the SCI subject, ROIs LFC, LCP, MCP, and RCP also showed relatively higher weights than for the healthy control subject, while the remaining ROIs have low weights for both subjects. Clearly, the cortical representation for the gait movements was more compact and strong for the able-bodied subject than the SCI user. These results demonstrate that MKL can be efficiently used to infer the importance of different groups of features and thus suggest different roles in the representation of gait for different scalp brain areas.

**Figure 5:**
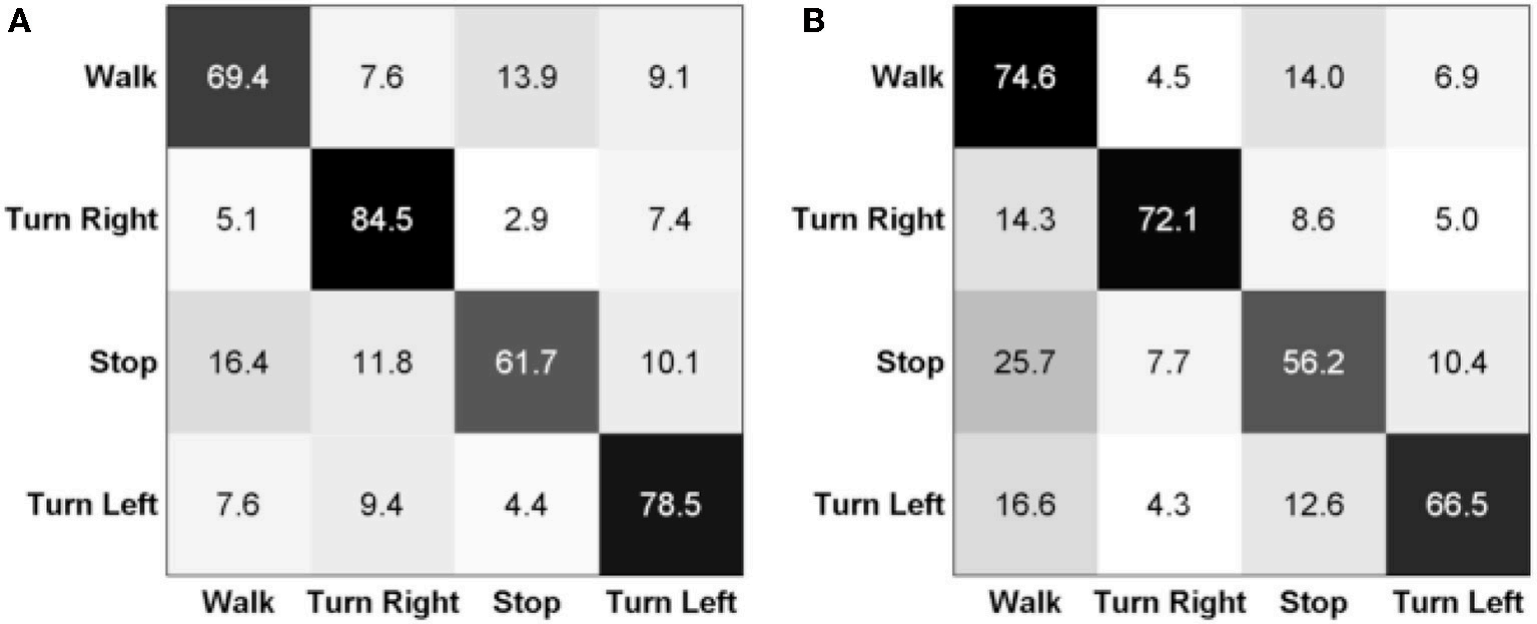
**Confusion matrices (%) for (A)** able-bodied subject and **(B)** SCI subject in Task 1.

Further, we give some insights of the class-wise results regarding different movement intentions. We show the confusion matrices in terms of class-wise accuracies and misclassification rates in Figure 5. Generally, the stop intention is the most difficult to decode—it was misclassified as walking forward in many situations. Turning right always has a high accuracy for both able-bodied and SCI subjects compared to the other classes. We note that all class-wise accuracies are above channel level—which is 25% for this problem.

### 3.2. Two-class, multiple sessions classification results

**Figure 6:**
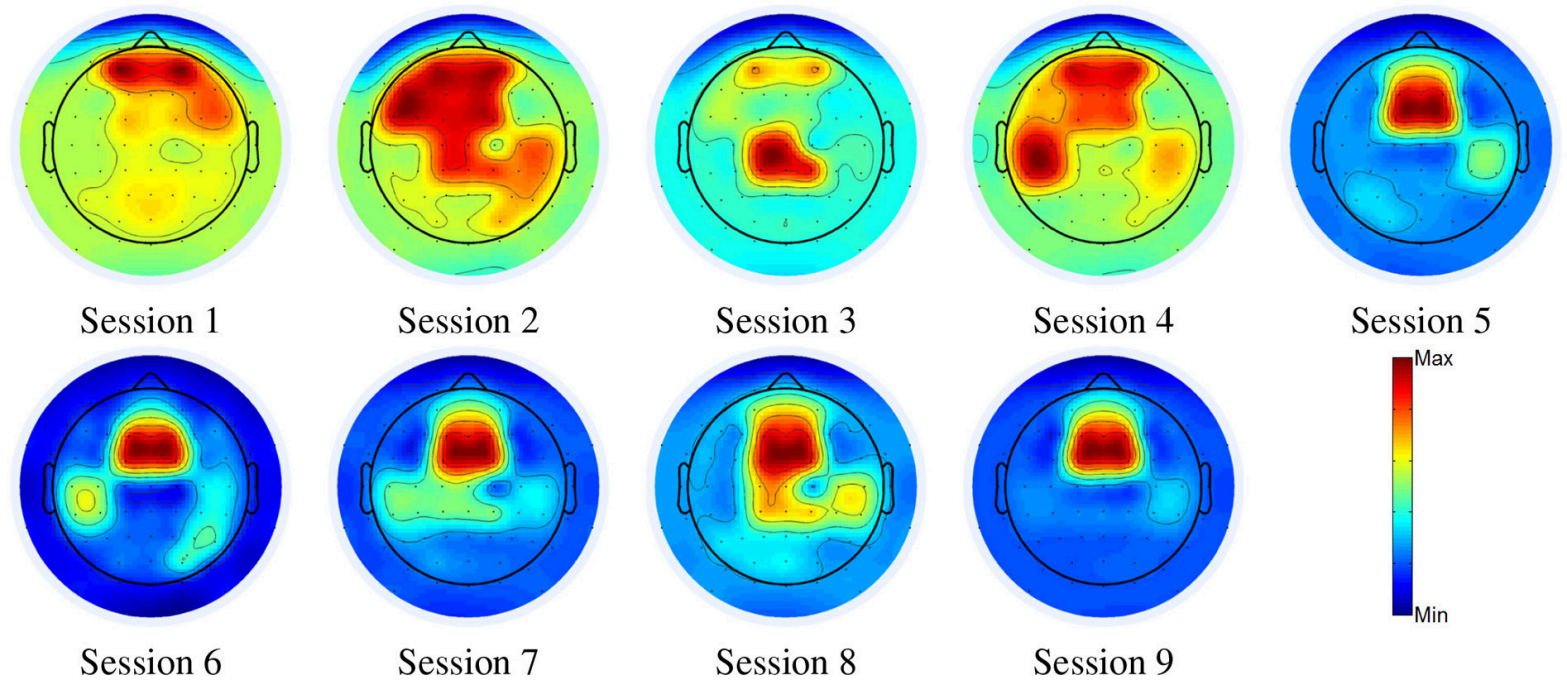
**Scalp maps of weights along 9 sessions for the able-bodied subject in Task 2**.

**Figure 7:**
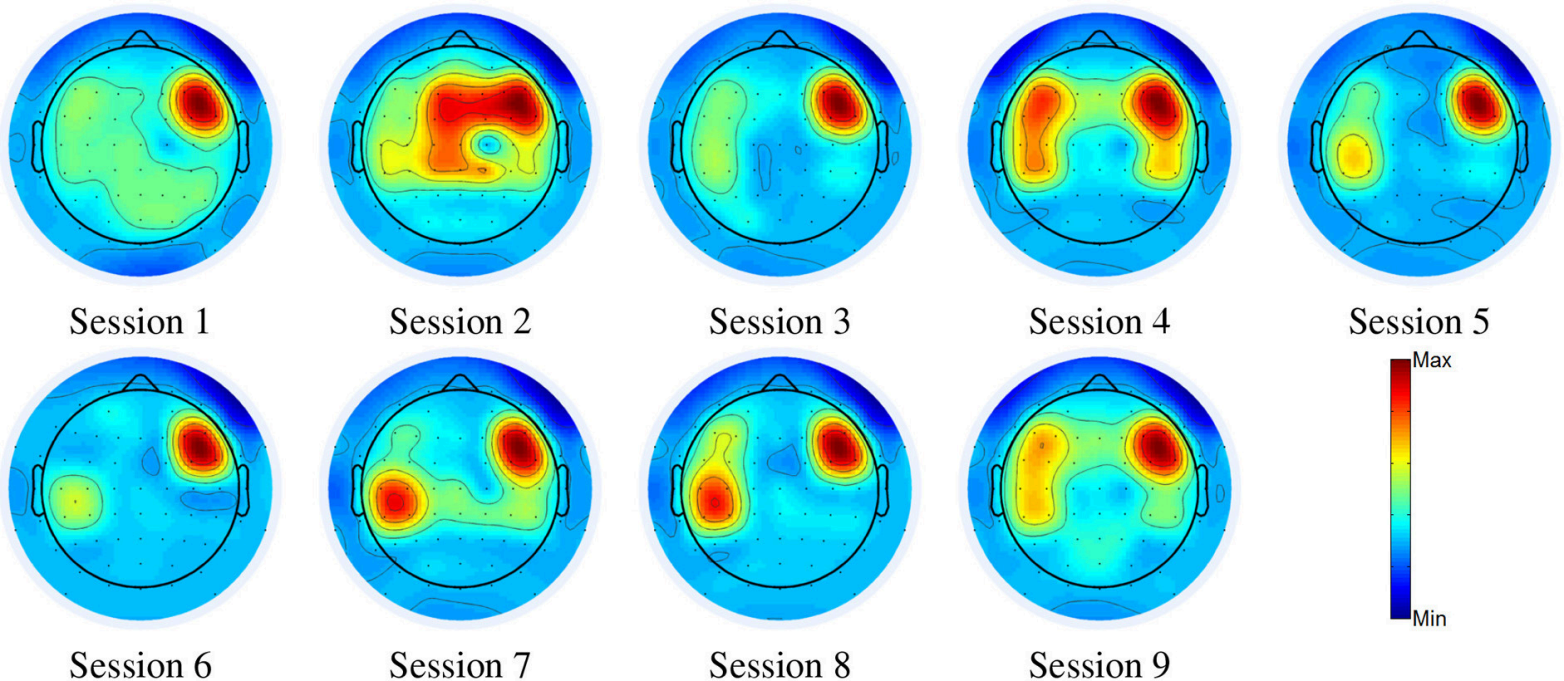
**Scalp maps of weights along 9 sessions for the SCI subject in Task 2**.

Second, we conducted a longitudinal experiment for the two-class (i.e., walk and stop) classification problem. We quantified electrode relevance changes across sessions to examine neural signatures that may indicate the cortical plasticity triggered by BMI use. We first plot the weight changes along 9 sessions over a period of 30 days for the able-bodied (a different subject as in task 1) and the SCI subjects (the same subject as in task 1) in Figures 6, 7, respectively. As depicted in the scalp maps, the weights change dramatically in the first several daily sessions, while becoming more stable in the later sessions. Similar to the previous results, the frontal scalp regions get the highest weights among all ROIs after training the user to control the exoskeleton for several sessions. Specially, for the SCI subject, RFC (ROI 4) has the highest weight, while LCP (ROI 5) also has relative high weight. For the able-bodied subject, the final important region is determined as MFC (ROI 3). Thus, the SCI subject used different brain regions to operate the BMI system when compared with the able-bodied user.

**Figure 8:**
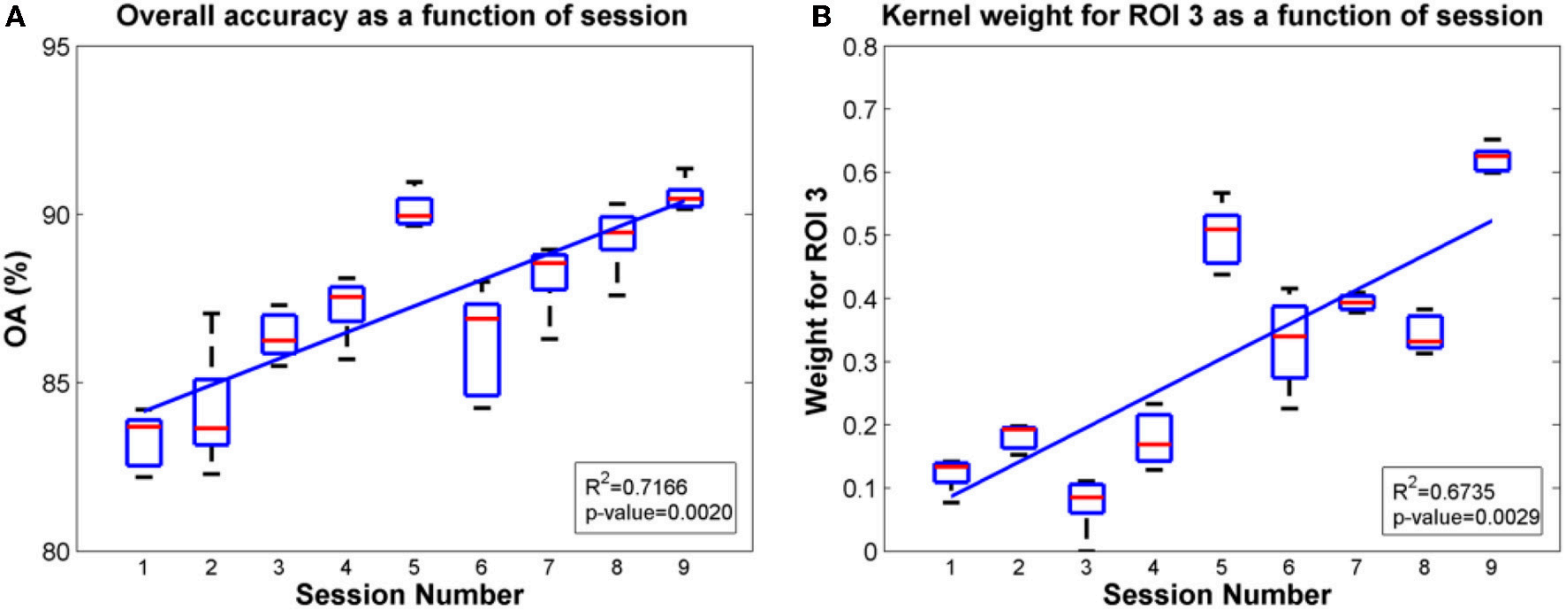
**Plots of overall accuracy and kernel weight for ROI 3 as a function of session for the able-bodied subject in Task 2**. **(A)** Overall accuracy as a function of session. **(B)** Kernel weight for ROI 3 as a function of session.

**Figure 9:**
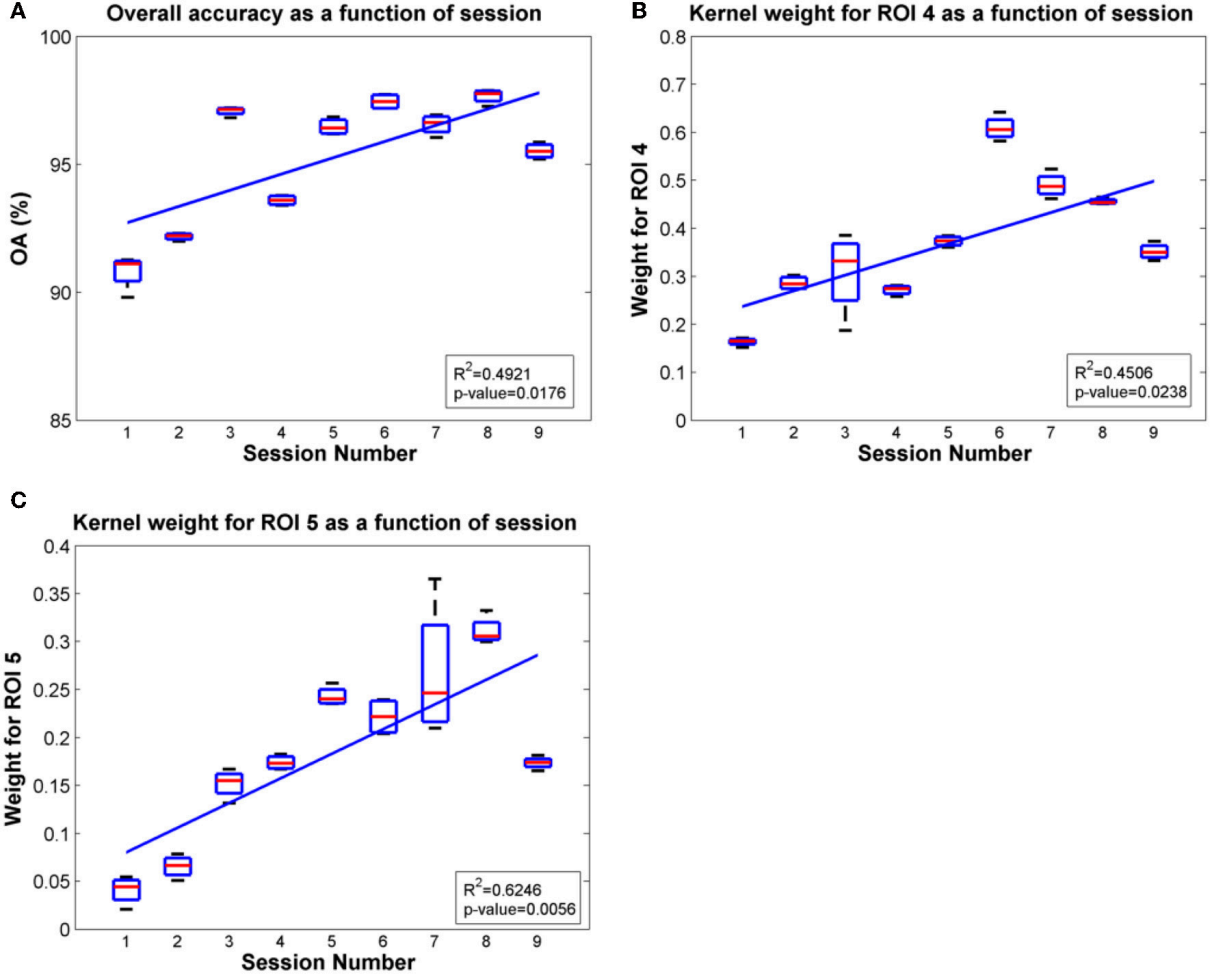
**Plots of overall accuracy and kernel weight for ROI 4 and ROI 5 as a function of session for the SCI subject in Task 2**. **(A)** Overall accuracy as a function of session. **(B)** Kernel weight for ROI 4 as a function of session. **(C)** Kernel weight for ROI 5 as a function of session.

Since ROIs 4, 5 and ROI 3 were determined as the most significant regions for the SCI subject and the able-bodied subject, respectively, for classification of gait states, we further evaluated the overall accuracy and kernel weight for these ROIs as a function of session. The linear fit of the relations between overall accuracy (or weights for the selected ROI) and daily BMI sessions are shown in Figures 8, 9 for the SCI subject and the able-bodied subject, respectively. From the results, we can see the classification accuracy generally increases as a function of session. At the same time, the weight for ROI 4, 5 (ROI 3) also showed a trend of increasing along the session over a period of 30 days. We calculated the *R*^2^ and *p*-value as a indicator of how well the data fitted to the regression line and the significance of the results to the hypothesis, respectively.

## 4. Discussion

Previous study (Kilicarslan et al., 2013) has shown the feasibility of classifying user movement intentions using EEG signals in the delta band (0.1–2 Hz) based on a GMM classifier and achieved a high offline evaluation accuracies for the 3-class tasks. In this study, we extend the study of intended motions from three classes to four classes and set the research as a longitudinal study. The experimental results demonstrate that by properly weighting the importance of the features, MKL can be used as an efficient decoder to predict user's movement intentions. For the tested subjects, the overall accuracies reached above 90% for the two-class classification task and above 65% for the more complicated four-class classification task. Compared to some commonly used machine learning approaches (i.e., Bayesian classifiers, LDA, SVM) in BMI, MKL has the following advantages (1) Unlike the LDA and Bayesian classifiers, MKL does not need to make assumptions on the data distribution. MKL is a member of kernel learning methods, which utilizes a linear combination of kernels and transforms the original data into an appropriate (kernel) feature space. Thus, all beneficial properties (e.g., optimality) of linear classifiers are maintained, while MKL is also efficient when the data are non-linear in the input space. (2) MKL is a robust learning method in the high dimensional space (Bach, 2008). In Kilicarslan et al. (2013), a sliding window was used on all 64 channels to extract features, it resulted in a 1,280 dimensional space, and a dimensionality reduction technique was required to reduce the dimension of the data decoding by GMM. Similar to the feature extraction step in Kilicarslan et al. (2013), we applied a 400 ms sliding window to extract the EEG delta band amplitude as input to the classifier. Differently, we divided the channels into different groups and extract features from each group. Thus, the resulting dimension for each group of data is at most 240, which is much lower than 1,280, and a dimensionality reduction method is not necessary for classification by MKL. (3) MKL can also be used to infer the importance of different groups of features, which is not feasible in other machine learning methods. The weight for each group is initialized uniformly at the beginning and optimized during the gradient descent in the MKL algorithm. MKL ranks sets of features corresponding to the meaningful features for solving the classification problem, and the results indicate importance in the representation of movement for different scalp brain areas.

Comparing the results from the SCI subject and the able-bodied subject, we observe the most important brain region changing from the midline fronto-central to the right fronto-central in both tasks. This could be due to the loss of brain cells and degraded cerebral cortex dynamics or lack of afferent input after spinal cord injury. For example, changes in movement-related cortical potentials have been noted after SCI and correlated with the severity of the injury (Boord et al., 2008; Gourab and Schmit, 2010). Moreover, altered spontaneous neuronal activity following SCI has been characterized by a shift in the dominant spectral power peak toward lower frequencies, including in primary and secondary somatosensory cortices (Tran et al., 2004; Sarnthein et al., 2006). Noted that the number of subjects participated in the study was limited, there might be individual variations on the classification results. However, as the classification results were based on standard cross-validation procedure, we believe the proposed approach and model can be generalized to other subjects.

In the longitudinal experiments, we found that the subjects were adapting to the BMI gait task in the first several sessions, so that the brain regions used for neural classification were not stable, which was reflected in the moderate classification performance. After several sessions of training, as the subjects learned to control the exoskeleton for movement, we observed the channels employed for movement classification converged to specific regions—the midline fronto-central areas for the able-bodied subject and the right fronto-central/left centro-parietal areas for the SCI user. In addition, the classification accuracy generally increased along session, and interestingly the weights for the important regions also increased. This demonstrates the cortical plasticity triggered by the BMI use, as the user gradually learns to control the exoskeleton for movement.

## 5. Conclusion

In this paper, we presented the feasibility of simultaneously classifying the pattern of user's internal gait states from the EEG signals and learning the relative importance of different scalp brain areas based on the MKL algorithm. The MKL has the advantages of learning the classifier and the optimal kernel weights simultaneously. We investigated these properties and applied the MKL classifier to infer the relative importance of different groups of features (different sources of information) in a BMI application to classify one's motion intention from the EEG signals. The experimental results demonstrated that the frontal/fronto-central regions were the most important regions for classifying gait states of the tested subjects, which is consistent with the brain regions hypothesized to be involved in the control of lower-limb movements. By comparing the results from the SCI subject and the able-bodied subject, the important regions were observed to change, which could be due to the loss of brain cells and degraded cerebral cortex dynamics or lack of afferent input after spinal cord injury. In the longitudinal experiment, while the user learned to control the exoskeleton for movement over multiple sessions, the classification accuracy increased and the weights for important regions stabilized. These findings suggest the cortical plasticity triggered by the BMI use, which will be investigated further in the future study.

## Author contributions

YZ developed the learning method, processed and analyzed the data, and wrote the manuscript. SP supervised development of work, helped in data interpretation and manuscript edit. AK helped to acquire and interpret the data. JC supervised development of work, helped in data interpretation and manuscript edit and evaluation.

## Funding

This work was supported in part by the NIH Award R01 NS075889, Mission Connect—A TIRR Foundation.

### Conflict of interest statement

The authors declare that the research was conducted in the absence of any commercial or financial relationships that could be construed as a potential conflict of interest.
